# The Trial of Robert Pate, or the Plea of Lunacy

**Published:** 1850-10-01

**Authors:** 


### Art. II.—THE TRIAL OF ROBERT PATE.

Since the publication of the last Number of our Journal, a trial of great importance has taken place, involving the elucidation of points of interest to all medical jurists and psychologists. A man, who once had the honour of holding her Majesty's Commission, has been arraigned at the Central Criminal Court for the commission of a most cowardly assault upon the Queen. In one of the most public thoroughfares in the metropolis, in the open face of day, and whilst surrounded by her children a person, having the exterior of a gentleman, had the audacity to strike her Majesty with a cane across the face with sufficient severity to leave behind perceptible marks of his brutal and cruel violence! At the trial, an attempt was made to obtain this man's acquittal on the ground of insanity; but the judge and jury indignantly repudiated the plea, and Pate was accordingly sentenced to seven years transportation! It is our wish to direct the particular attention of our readers to this case. In the first, and in subsequent numbers of this Journal, we have fully discussed the question of criminal insanity. It will therefore be unnecessary for us to enter again into any detailed exposition of the principles which, in our judgment, should guide those entrusted with the grave responsibility of deciding as to the existence of insanity in any given case of crime. The plea of insanity is one of the most important that can be urged in a court of justice in extenuation of the criminal acts of individuals. It should never be advanced except in clear and obvious cases, where little or no doubt can be entertained as to the existence, not only of derangement of mind, but of derangement of such a *kind,* and to such a *degree,* as to justify the immediate admission of the fact, and the necessary and consequent acquittal of the prisoner. The greatest caution should be exercised in the use of the plea, and medical men especially engaged in the study of mental diseases ought, above all others, to be particularly upon their guard in giving evidence in these cases, exhibiting a jealousy lest their opinions should be used for the purpose of shielding great criminals from the legal penalties awarded as a punishment for serious inroads upon the recognised laws which bind society together. In matters of this kind, the public and profession naturally place a high value upon the experience and judgment of men whose peculiar studies and opportunities of obtaining a practical insight into the morbid phenomena of the human mind, specially qualify them for the elucidation of difficult points connected with criminal insanity. If it be found that the most eminent of British psychologists are disposed to stretch this important and valuable plea beyond its legitimate limits, then a reaction will certainly take place in the public mind, and these questions will be decided in our courts of law without having recourse to medical evidence, and points of lunacy will be left altogether to the judgment and common sense of an English judge and jury. That a disposition of this kind unfortunately exists, is very evident.* This feeling, we lament to say, is not confined to those organs supposed to represent public opinion, but medical journalists have joined in the hue and cry against the abuse of the plea, and in doing so have, we think, most unjustifiably, endeavoured to undervalue the evidence of those usually called upon, in consequence of their enlarged experience, to substantiate it in our criminal courts. These are important signs of the times; and although they ought not, in the slightest degree, to deter the psychologist from the manly, independent, and honest expression of his scientific opinion, they certainly should induce him to act with great caution and judgment when called upon to give evidence in any cases involving the intricate, subtle, and important questions of crime and insanity. Of the value of the medical evidence of those whose specialty it is to investigate the wonderful changes which disease effects in the human mind, there

* We cite the following passage from the *Morning Post* of July 13, as illustrative of the attacks to which medical men are subject who give evidence in these cases:—"Had the jury suffered themelves to be swayed from the legal as well as common sense view of the question by the perverted metaphysical refinements of Drs. Conolly and Monro, the consequences of the course into which the mischievous guidance of those fanciful gentlemen would have led them must have been eventually most disastrous.

"Dr. Conolly appears to have devoted his attention so exclusively to the various shades of mental disease that his own understanding on these points has lost the power of discrimination, and he has refined away to such an extent all distinctive boundaries between the phases of progressive mental abberration, from occasional eccentricity to utter loss of reasoning power, that he can apparently no longer distinguish where absolute madness begins and moral and legal responsibility ceases. There are very few of our fellow-subjects, we suspect, who could get from Dr. Conolly a certificate of perfect sanity—very few who would not, *judice* Dr. Conolly, be properly located at Hanwell. In his view, all persons who ambitionless saunter along the path of life, without setting before them any especial object for attainment, exhibiting, with a feeble and indolent controlling will, occasional outbursts of passion or erratic impulses, are of unsound mind, and fit subjects for exemption from the ordinary consequences of the infraction of the laws by which the rest of society is bound.

"We believe we have not in the least degree strained the interpretation of the evidence given by Dr. Conolly, as reported on this occasion in the columns of our contemporaries, as well as in our own. Nay, Dr. Conolly goes even further than we have represented; he admits that Mr. Pate undoubtedly knew the distinction between a right and a wrong action, that he was well aware that it was wrong to strike the Queen, but still he comes to the conclusion that Mr. Pate is of unsound mind, because the action was apparently motiveless and the result of a strange uncontrollable impulse. Is it possible that Dr. Conolly, who has concentrated his attention for so many years on the diseases of the mind, is not aware that across the soundest minds flit occasionally wild desires, strange imaginings, and fantastic fancies, which the will feels a strange anxiety to yield to, with no greater motive than that of enjoying the pleasure of putting them in action, and which it is only restrained from doing, either by the dread of certain consequent human punishment, or the more powerful control of religious principles?"

cannot, among thinking, sensible, and unprejudiced men, be two opinions. It has been said that the psychologist sees insanity where the ordinary medical philosopher can recognise no shade of deranged mind, and that his natural bias is in favour of the existence of insanity in every case where the faintest suspicion has arisen as to its presence. This is foolish twaddle. Why should not the psychologist be capable of giving as honest an expression to his opinion as any other section of the medical profession? No one dreams of questioning the judgment or sagacity of a Bright, a Rees, or a Bird, when called upon to decide as to the pathological condition of the kidney, in any given case—no person would have the audacity to challenge the opinion of a Brodie, a Lawrence, or a Fergusson, on any matter involving a complex surgical point of practice; no person would have the presumption to whisper the semblance of a doubt as to the profound practical sagacity of a Seymour, Watson, Latham, or a Williams, in any medical case, requiring, for its successful elucidation, a mind enriched by the vast resources of medical art and science; no sane person could doubt the extreme value of the opinions of a Christison or a Taylor on a point of medical jurisprudence; and yet, if we were to carry out the principle advocated by some who have undertaken to enlighten the profession upon these abstruse points, no greater weight or authority is to be attached to the opinion of these distinguished men than we should extend to less informed and cultivated minds, who have not enjoyed a tithe of the opportunities of acquiring a knowledge of the more profound branches of pathology and therapeutics possessed by those who deservedly are placed, by almost common consent, in the foremost ranks of the profession. The psychologist, whatever may be the natural and original strength of his understanding, the acuteness of his powers of observation, perception, and reflection, and the extent of his opportunities of acquiring a minute knowledge of the subtle characteristics of disordered mind, in all its erratic forms, with the best intentions to arrive at the truth is liable to errors of judgment; but to state that his opinion is of no more value and importance in the elucidation of the action of a mind clouded by disease, or thought to be so, than that of a practitioner whose attention has never been directed to the study of insanity, and whose opportunities of becoming practically acquainted with the habits of the insane have been less than nothing, is to state what is intensely absurd, and utterly at variance with the common sense of the profession. Such attempts to distort the truth, and to pander to low and vulgar prejudices, are unbecoming the members of a liberal profession. The men who thus endeavour to lower, in public and professional estimation, the opinions, learning, and labours of the British psychologist, might as well think of levelling St. Paul's Cathedral to the ground, by butting their empty heads against its porticoes. Whilst we give this warm expression to our opinion, we nevertheless think it our duty, as public journalists, to caution this section of our profession against a lax use of the plea of insanity; we must not give our enemies *in* and *out* of the profession the slightest excuse for these illiberal and unjust attacks upon a body of men deserving, in every point of view, the confidence and support of all enlightened men. Having premised these few observations, we would address ourselves to the special subject of this paper. In another portion of the journal, we have printed, *in extenso,* the trial of Pate. Notwithstanding the respectability of the medical and general evidence adduced, we are compelled, by a stern sense of duty, to express our concurrence in the verdict of the jury. We do not belong to that section of psychologists, who maintain that in every case the plea of insanity should be held as conclusive and valid, without any reference to the kind and degree of the mental disturbance which has been supposed to give rise to the criminal act. This is no recently formed opinion. In a work published in 1843, we have placed upon record similar views.* We then observed:—

"In a previous part of this work, I have ventured to express an opinion, opposed, I know, to that generally entertained by many who have written on this subject. I again repeat, that I am not prepared to give an unqualified assent to the dogma, that in every case of mental derangement,—without any reference to its degree or character,—ought the person to be screened from the penalty awarded by the laws for criminal offences. I am ready to admit, that if insanity be clearly established to exist, a *primâ facie* case is made out in favour of the prisoner; but that because a person may be proved to be strange and wayward in his character, to fancy himself a beggar when he may have the wealth of a Crœsus, or to be ill when he is in the buoyancy of health—to believe that such a person ought, of necessity, to be exonerated from all responsibility, is a doctrine as unphilosophical and untenable as it is opposed to the safety and well-being of society."

Our opportunities of extended experience have been great since the publication of this opinion. The more we have seen of insanity, particularly among criminals, the stronger are our convictions that this is the sound, the safe, and philosophical view of the question of derangement of mind complicated with crime. If the extreme view of the subject is recognised, and acted upon, society would not be safe; if every shade of disturbed mind, if any amount of eccentricity, even when evidently associated with unhealthy cerebral organization, or even incipient insanity, is to shield persons from the just punishment awarded for offences against life and property, we might be justified in closing the

* The Plea of Insanity in Criminal Cases. 1843. By Forbes Winslow, M.D.

doors of our criminal courts, and superannuating the judges who are intrusted with the administration of the laws! If we carried out this principle to its full extent, many great criminals would elude the hands of justice, and escape the just punishment awarded for crime.

As we have stated in a previous number of this Journal, a sufficiently just and scientific distinction is not drawn between the actions of a *naturally eccentric, ill-regulated, perverse,* and *wicked* mind, and the mental disturbance, perverseness, caprice, and viciousness which *are clearly traceable to disordered mind, consequent upon physical disease.* There is a *natural* and *healthy* eccentricity, caprice, distorted and unnatural affection, vice, and disposition to acts of violence and cruelty, which are quite independent of that disturbance of the current of thought and perversion of the will, the clear and evident consequences of physical disease or disorder resulting in a morbid action of the faculties of the understanding. As a man may have a natural *physical,* so he may have a connate *mental* defect, apart altogether from disease of the mind, and which undoubtedly places him among the responsible portion of society. Natural eccentricity, oddity, and perversion of the mind are sometimes confounded with the *products of disease;* and this has led to much misconception of opinion, useless disputation, errors of conduct and judgment. We see nothing in the case of Pate which removed him from the responsible class of society. We are ready to admit that a sufficient degree of eccentricity and irregularity of thought and action was established to justify the grave suspicion of his mental unsoundness, and entitled his case to serious inquiry; but that the history of the case, and the evidence adduced at the trial, justified the acquittal of the prisoner on the plea of insanity, we are not prepared for a single instant to admit.

"If we confine," says a contemporary,* "our attention to what transpired on the morning of the outrage, we shall discover nothing in the least degree indicating that he was insane. He left his home as usual; joined the crowd at Cambridge-gate as other people did; watched quietly for the approach of her Majesty's carriage; and accomplished the attack with great quickness and dexterity. When seized upon the spot, he was perfectly sensible of what he had done; he knew it was her Majesty whom he had struck; and there was not the least appearance of incoherency either in his manner or his observations. If we take the retrospect of his life, which was furnished during the trial, and ransack the incidents of his personal biography, we must still come to the same conclusion; true, he was eccentric enough, but not more so than other men, were we only able to observe the peculiarities which constantly occur in the solitary and private life of almost every individual. He lamented the death of his dog—we have seen a high-

* Medical Times.

minded and very illustrious professor shed tears over the death of a noble Newfoundland dog, which was wickedly and spitefully poisoned. He lamented the loss of his horses—we have seen young men at College half maniacal over a much less calamity befalling a favourite hunter. He shouted and sang aloud when he was in his bath—we were not many weeks ago at Brighton, and there heard an eminent Chancery barrister and a celebrated Italian *artist*, in the swimming bath, shouting and fighting with the watery element as if they were stark mad. He put whiskey and camphor into the water in which he bathed—an old woman's remedy, which many sensible persons adopt at different watering places, particularly in the north. He bought some nursery rhymes, and read them through—very lately we met a learned friend of ours, who had bought, for sixpence, a copy of Æsop's Fables, and was reading them with great delight. He walked about the streets in a hurried manner, switching his cane to and fro, and we are told looked wild—perhaps so, but he took care never to be run over, or to meet with any other accident. He ordered a cab-driver to come to his door daily at a given hour, and drive him over to Putney-heath, where he walked for about ten minutes among the furze—how many hundreds daily take a far more monotonous drive in Hyde park? Lastly, he had a dyspeptic attack, and talked about having bricks and stones in his stomach—but there is no evidence to show that these substantive expressions were meant to convey more than a description of the uneasy sensations he endured. Where, then, is the evidence of this Robert Pate's insanity? He discharged his duties in his regiment 'to the satisfaction of his superior officers, and was esteemed by them and everybody in the regiment.' At his lodgings, his conduct was exemplary—'he paid his accounts very regularly, put them away, and kept the receipts;' but notwithstanding all this, Dr. Conolly declares his opinion that this man was of unsound mind. 'I form this opinion,' he observes, 'from the conversations I myself have had with him, and from all the other facts I have heard, but principally from the former. *It seemed to me that he has a very small share of mental power, without object or ambition, and unfit for all the ordinary duties of life. In conversation he would undoubtedly know the distinction between a right and a wrong action, but I should say that he would be subject to sudden impulses of passion.*' We cannot conceive more inconclusive reasoning. Does it follow that because a man has a 'small share of mental power,' and is without 'object or ambition,' that therefore he is not amenable for his actions to the laws of society? The opinion of Dr. Conolly, that he was 'unfit for all the ordinary duties of life,' is directly at variance with the evidence to which we have referred. Dr. Conolly admits, that 'in conversation he would undoubtedly know the distinction between a right and a wrong action.' Why then should his conduct not have been governed by his perception of right and wrong, since it is admitted there was no lesion of his intellectual faculty? 'But I should say,' adds Dr. Conolly, 'that he would be subject to sudden impulses of passion.' So are men of evil disposition and bad moral habits; but surely this unhappy mental condition ought not to be allowed as an extenuation of crime. Dr. Monro states, that he had examined the prisoner five times since his committal, and was of opinion that he was of unsound mind; but his reasons for coming to that conclusion do not appear to have been elicited upon cross-examination."

Again, another medical journalist* observes—

"We do not remember to have met with a case in which the plea of insanity was advanced upon weaker grounds than in this. Admitting Dr. Conolly and Dr. Monro to be correct in their opinion that the prisoner was of unsound mind, it is clear from the present state of the law, that his unsoundness or weakness of mind had not reached that degree to render him irresponsible for offences committed against others. Mr. Pate was not so mentally unsound as to be unsusceptible of correction by punishment; and the verdict returned against him will, we think, have the good effect of preventing others in the same weak mental condition from committing an assault upon her Majesty. If the moral powers of such persons are not sufficient to control their criminal impulses, the recollection that a similar act was punished by seven years' transportation, may suffice to restrain the arm thus wantonly raised against the Sovereign."

The law, as laid down by the judges in the House of Lords, when this important question was brought under their consideration, distinctly asserts that no defence on the ground of insanity is to be considered valid, unless it can be clearly established that the person alleged to be insane is unacquainted with the distinction between right and wrong. If he be conscious that he is violating the law, and has sufficient capacity to appreciate his relative position to the law of the land, he is justly held amenable for his conduct. We must have some *test* of responsibility, and this, by universal consent, is considered by those who have the administration of justice, the best standard that could be devised. It is the only test (however imperfect it may be) of the plea of insanity recognised by the law of England. As both of the medical gentlemen who were examined distinctly affirmed that Pate knew the distinction between right and wrong, the verdict was in strict uniformity with the law, as expounded by all our great judges. But, apart from the strict legal and technical view of the case, we have no hesitation in stating that the jury came to a just decision. The medical evidence certainly did not warrant any other result.

It should never be forgotten that there is always afloat upon the surface of society a large body of strange, wayward, intemperate, eccentric, half-cracked, and half-educated persons, individuals who delight in deviating from the ordinary and recognised mode of thinking and acting, who are subject to acts of great caprice—naturally impulsive in all their actions—inflated with ideas of their own importance—

* Medical Gazette.

subject to uncontrollable and violent passion, (because they have never been taught the art of *self-control,*) vicious, depraved, sensual, and devilish, ever anxious to creep out of their natural insignificance, and to make themselves the subjects of public notoriety. This constitutes one of the most dangerous sections of society outside the walls of Bethlem and St. Luke's. They endanger the public peace, give rise to great anxiety on the part of their relatives, and engender in private families an immense amount of heart-burning and unhappiness.

Again, there is a large class of persons who, from want of education, from the habit of pandering to vicious inclinations, or owing to the existence of some natural constitutional defect, or from being exposed to the depressing and exciting emotions, are brought very closely within the domain of actual insanity, and are seen hovering between the confines of sanity and mental derangement. Both these classes are, in the strict signification of the term, capable of exercising self-control, of knowing the distinction between right and wrong, and therefore are to be viewed as responsible agents. We cannot contemplate without feelings of serious apprehension the inevitable consequences of an indiscriminate and unscientific admission of the plea of insanity upon the minds of the two classes previously referred to. If they are led to suppose that it is only necessary to prove the existence of eccentricity, extreme capriciousness, great deviation from the ordinary habits of life, in order to escape the criminality of their actions, we feel assured that an important controlling influence would be removed from a considerable section of society. These persons must be taught the necessity of *self-control;* that their eccentricity and tendency to acts of violence, whatever may be the motive that urges them on, will not protect them from punishment.

The power of self-control is, in many instances, weakened, or altogether lost, by a *voluntary indulgence in a train of thought, which it was the duty of the individual in the first instance to resolutely battle with, control, and subdue.* Insanity is thus often *self-created:*—

"*King Phil.* You are as fond of grief as of your child.
*Constance.* Grief fills the room up of my absent child,
Lies in his bed, walks up and down with me,
Puts on his pretty looks, repeats his words,
Remembers me of all his gracious parts,
Stuffs out his vacant garments with his form;
Then have I reason to be fond of grief."—*King John.*

The state to which grief has reduced the mind of Lady Constance frequently precedes insanity, and is, to a certain degree, a voluntary act of the mind; the intellect retaining its sanity conjointly with the power of volition. Directly the will ceases to control the thoughts and emotions, the understanding loses that balancing agent which preserves it in a state of healthy equilibrium.

A man indulges in a depraved course of conduct; harbours and encourages a vicious current of ideas, his actions often corresponding with the unfortunate condition of his mind and feelings, until all power of volition becomes suspended, and he is actually reduced to a state of lunacy.* A person, perhaps for some real cause, feels a degree of animosity towards a particular individual who has injured him. Instead of making an effort to conquer this feeling, he allows,—in fact, forces—his mind to dwell upon it; the idea pursues him in all his walks; haunts him in his waking thoughts; exercises its ascendancy over him during the slumbers of night. The mind eventually becomes so absorbed in the idea that the bitter angry feeling, which, at the *first onset,* was insignificant and controllable, takes full possession of the mind, and influences and distorts every thought and action. The mind soon becomes diseased, the insanity manifesting itself *in an exaggerated and extravagant conception of an idea which had some semblance of truth for its existence.* The *self-created* morbid idea may thus obtain a complete mastery over the mind, and lead to the commission of crime.†

If self-control is to be exercised with any great advantage, it is certainly in the earlier or incipient stages of disordered mind; at this period *it is possible, even when insanity has clearly commenced its inroads upon the intellect, to subdue the morbid thoughts, feelings, and impulses, by a resolute and determined effort of the will.* It may be said that the disorder of the mind, which is so often palpable in the incipient stage, is not insanity in the proper acceptation of the term; that it is not entitled to this appellation until the power of volition is suspended. But this is not altogether a sound view of the case. We are willing to admit the extreme difficulty—nay, the impossibility—of drawing the line of demarcation between sanity and insanity, responsibility and irresponsibility. Who will venture to point out the neutral ground? Where is, we might exclaim with a distinguished historian,

* "The indulgence of violent emotions," says Dr. Conolly, "is singularly detrimental to the human understanding—and it is to be presumed, that the unmeasured emotions of insanity are sometimes perpetuated in consequence of the disorder of the brain, originally induced by their violence. A man is at first only irritable, but gives way to his irritability. Whatever temporarily interferes with his bodily or mental functions, reproduces the disposition to be irritated, and circumstances are never wanting to act upon this disposition till it becomes disease. The state of the brain, or part of the brain, which is produced whenever the feeling of irritation is renewed, is more easily induced at each renewal, and concurs with the moral habit to bring on the paroxysm on any slight occasion—other vehement emotions and passions effect the same disorder of the mind."

† Ethical philosophers have maintained that man is responsible even for his *dreams.* It is argued that the train of ideas occurring during the period of sleep is palpably influenced by the *voluntary* thoughts during the day.

when discussing the propriety of the resistance made to the tyranny of James II.,*—where is the frontier where virtue and vice fade into each other? Who has ever been able to define the exact boundary between courage and rashness, between prudence and cowardice, between frugality and avarice, between liberality and prodigality? A good action is not distinguished from a bad action by marks so plain as those which distinguish a hexagon from a square. Carrying out this view of the case, we should ask who would be so bold as to define the boundaries of vice and insanity? The extreme difficulty of the subject ought to humble human pride, and make even the most confident and experienced among our psychologists cautious in deciding, without careful investigation, on matters so subtle, mysterious, and complex.

As great power of self-control manifestly exists in the earlier periods of mental derangement, and as many cases of incipient insanity are cured by the determined efforts of the individual to keep in abeyance his disposition to engender a morbid train of thought, how important does it become that those so unhappily situated should be assisted in their efforts to ward off mental disease? How is this to be effected? Not, certainly, by withdrawing from them the *moral and controlling influence of the law.* The majesty of justice must not only be vindicated, but the healthy influence of the law be fully maintained. *The eccentric, the wayward, the capricious, the vicious, the habitually intemperate, violent, impulsive, and passionate man, and even those more closely bordering upon insanity, must be taught, in clear and unmistakeable language, that the tribunals of the country hold them responsible for their actions; that they cannot with safety or impunity outrage the laws or decencies of society, or shield themselves from punishment; that the law considers them responsible for their actions.* If this idea had been fully impressed upon the mind of Pate, would he have committed so gross an assault upon her Majesty? We doubt it. We are not justified, as members of a Christian community, as citizens anxious to hold inviolable the laws which bind and protect society, in advocating a doctrine calculated to give full play and exercise to the unbridled passions and impulses of a number of weak-minded, half-idiotic, morbidly-conceited persons, who are constantly swimming upon the surface of every great community, and ever anxious to make themselves objects of public notoriety, without any regard to the means by which they effect their object. Take for illustration the following fact:—

"A person of the name of *Edwin Bates,* who stated that he was an artist by profession, was charged at the Bow-street police-office with having sent, with the view of extorting money, threatening letters to His Royal Highness Prince Albert. Mr. Jardine, the magistrate,

* Mr. Macaulay.

heard the case, the prosecution being conducted by Mr. Raven, of the Treasury. It appears that the prisoner addressed a number of letters to His Royal Highness, soliciting pecuniary assistance, with the view of relieving him from alleged railway losses. Some of the letters contained expressions implying that it was the intention of the writer to do His Royal Highness some bodily harm, unless his letters and solicitations were satisfactorily replied to. The letters were shown to the prisoner, and he immediately admitted the authorship. In his defence, Edwin Bates, the prisoner, accused His Royal Highness of unkindness. He said that he had applied to other noblemen and gentlemen, who had responded kindly to his appeal for pecuniary assistance. Prince Albert declined to aid him. In pleading for a mitigation of his sentence, he observed—'If you make an example of me, you will deprive me of all the subscriptions. I hope your worship will consider the state of my mind at the time of my writing these letters, and put a favourable construction upon them, for I can assure you I have no hostile feelings towards the royal family, and they have none towards me. You have only to send to Dr. Monro to learn the state of my mind. When I was tried for felony, thirteen years ago, I was considered insane. I do not wish to speak of my own weakness; but it is well known that I am not in my right mind.'"*

It is a fact well known to those who have had much to do with the insane, that the power of self-control is not altogether lost even in persons whose kind and degree of mental derangement are such as to justify their forcible separation from home and society, and confinement in an asylum. We have witnessed in very obvious cases of lunacy the exercise, for a purpose, of great—even extraordinary—powers of self-control. To say that lunatics are not amenable to many of the influences that regulate the actions of sane persons, is to assert what is not the fact. Of course, the greater proportion of the insane are with extreme difficulty controlled, and are incapable of controlling themselves; but we are disposed to believe that even this view of the case may be exaggerated. It is the duty of the scientific and philosophic physician intrusted with the care of the insane, to develop in his patients the habit of self-control, and much of his comfort and success will depend upon the extent to which he is enabled to effect this desirable object.* If positive lunatics are to be influenced by expectation of rewards and punishments, (and they certainly are,) it is not a great stretch of fancy to suppose that those, be they insane, or bordering upon insanity, who are allowed to be at large and mix in society, are susceptible of the same moral influences; and if they do not possess the

* Times, July 23.

† "It is," says Dr. Prichard, "a great error to suppose that lunatics are not susceptible of moral discipline, or capable of being brought under the control of motives similar to those which govern the actions of other persons. It is very possible to subject them to such a rule, and this constitutes, indeed, a very important and essential part of the means of cure."

power, they ought to be taught the necessity of bringing their thoughts and actions within the range of sound reason and judgment. We are anxious that our views on this important subject should not be open to misconception or be misconstrued. God forbid that we should stand forward as apologists for those who maintain, even in these enlightened days, that the criminal lunatic should be subject to the extreme penalty of the law! The advocates of such a doctrine will find no mercy at our hands. We question the propriety of even using the word punishment in association with the really insane. If it be found necessary, in order to bring them within proper control and discipline, to subject patients afflicted with derangement of mind to certain stringent regulations, it ought only to be considered as a part of a system of rational, humane, and philosophic moral treatment. *In every criminal case where the question of responsibility arises in the course of judicial inquiry,* IF IT BE POSSIBLE TO ESTABLISH ANY DEGREE OF POSITIVE INSANITY, IT SHOULD ALWAYS BE VIEWED AS A VALID PLEA FOR A CONSIDERABLE MITIGATION OF PUNISHMENT, AND AS PRIMÂ FACIE EVIDENCE IN FAVOUR OF THE PRISONER, AND IN NO CASE WHERE INSANITY CLEARLY EXISTS, (WITHOUT REGARD TO ITS NATURE AND AMOUNT,) OUGHT THE EXTREME PENALTY OF THE LAW TO BE INFLICTED. We feel convinced that the principle just enunciated is the most safe and humane one to act upon; it does not protect the pseudo-lunatic from just punishment, and it is conservative in its operation upon society as well as upon those who are really under the influence of diseased mind, and are susceptible of being controlled. Two great objects will be thus obtained. We feel assured that this view of the question will meet with the support of a large and influential section of the profession, as well as of that portion of the public press which is ever ready to watch with a jealous eye the evidence of the psychologist in favour of the extenuation of crime on the ground of insanity.

---

